# Dose-Response Relationship between Exercise Duration and Executive Function in Older Adults

**DOI:** 10.3390/jcm7090279

**Published:** 2018-09-13

**Authors:** Feng-Tzu Chen, Jennifer L. Etnier, Chih-Han Wu, Yu-Min Cho, Tsung-Min Hung, Yu-Kai Chang

**Affiliations:** 1 Department of Physical Education, National Taiwan Normal University, Taipei 10610, Taiwan; alexnewtaipei@gmail.com; 2 Department of Kinesiology, University of North Carolina at Greensboro, Greensboro, NC 27413, USA; jletnier@uncg.edu; 3 Graduate Institute of Athletics and Coaching Science, National Taiwan Sport University, Taoyuan 33301, Taiwan; wu75757979@hotmail.com; 4 Center for East-West Medicine, University of California, Los Angeles, CA 90095, USA; cho.yumin@gmail.com

**Keywords:** dose-response relationship, executive function, exercise prescription, task switching

## Abstract

This study aimed to determine the dose-response relationship between exercise duration and task switching in older adults. Acute moderate intensity aerobic exercise for 20 min resulted in shorter response times than control and 10-min sessions in the heterogeneous, non-switch, and switch conditions, but not in the homogeneous condition. Additionally, linear and cubic trends between exercise duration and global switching performance as well as local switching performance were revealed with faster times being predicted by longer duration exercise; however, the cubic relationship resulted in performance following the 45-min session being not significantly different from the other three sessions. Acute aerobic moderate intensity exercise for 20 min is an effective duration to improve task switching. Although a longer duration of exercise is not optimal for benefiting task switching, it does not harm task switching in older adults and hence may be of value for other health-related reasons.

## 1. Introduction

In many cases, lower cognitive function is associated with advancing age in adulthood, and age-associated cognitive impairment causes enormous personal and societal burdens [1,2]. While multiple aspects of cognitive function have been linked to aging, the cognitive domain of executive function is particularly relevant. Executive function describes complex and higher-order cognitive processes used to monitor, manage, and regulate a self-directed set of purpose-oriented actions to reach a desired goal [3]. Executive function is recognized as a crucial contributor to the maintenance of independent function in advancing age, and declines in executive function are predictive of mild cognitive impairment and dementia in later life [4,5]. Executive functions are largely controlled by frontal lobe activity [6], and there is evidence confirming that the frontal lobe is sensitive to advancing age [7,8]. It is important to recognize that engaging in “exogenous” challenges, such as physical exercise, can benefit executive function [9,10,11,12]. Indeed, even a single bout of exercise, known as “acute exercise”, has been shown to positively affect executive function [13,14,15]. While this positive effect has been demonstrated in aged adults [14,16,17], there are two important considerations relative to this relationship that have not as yet been explored. 

First, executive function is a comprehensive term, but it is not a unitary/single construct. In other words, executive function describes higher order functions that include inhibition, updating/working memory, and shifting/task switching [18]. In previous research, the focus has largely been on inhibition and/or working memory [19,20,21,22], making a good understanding of the effects of a single session of exercise on other aspects of executive function unknown. In particular, task switching is the focus of this study because of several theoretical considerations. Task switching reflects executive control processes associated with the flexibility to shift between different tasks or mental sets and has become a primary metric for studies on aging [23,24]. Furthermore, although task switching tasks may also involve inhibition and working memory aspects of executive function [25], task switching can be distinguished from other types of executive function from neural perspectives [18]. For example, task shifting is associated with brain atrophy in the bilateral frontal cortex, whereas inhibition is located in the right inferior frontal gyrus and working memory is distributed in the left anterior cingulate gyrus, left premotor cortex, and right inferior frontal gyrus in older adults with mild cognitive impairment [26].

Acute exercise has been shown to induce activation in specific brain regions associated with executive function, including the dorsolateral prefrontal cortex that has been previously linked to task switching [27]. This suggest a potential connection between acute exercise and task switching. In the few studies that have employed task switching, acute exercise has been shown to benefit task-switching in young adults [28] and in a sample of young and older athletes [17]. However, it remains unclear whether the improved task switching performance resulting from acute exercise extends to the general older population.

Additionally, although a considerable corpus of research has shown that acute aerobic exercise has a positive effect on executive function [13,14,21], it remains unclear as to which particular “dose” of exercise is needed to achieve maximum benefits. According to the guidelines of the American College of Sports Medicine (ACSM), considerations for acute exercise beyond mode (e.g., bicycle, treadmill, resistance exercise) include intensity and duration. Studies testing dose-response relationships provide important guidance for exercise prescription [29]. To date, the focus in the literature has been on understanding the relationship between exercise intensity and cognitive performance. Through empirical studies and meta-analytic reviews, an inverted-U relationship has been observed, with moderate intensity typically resulting in better performance than low or high intensity exercise [13,30]. This finding is consistent with the classic arousal-performance hypothesis provided by Yerkes and Dodson [31]. However, to our knowledge, only one study has examined the dose-response relationship between exercise duration and executive function. Chang, Chu [32] tested the effects of moderate intensity aerobic exercise on Stroop task performance (a measure of inhibition) at several different durations (i.e., control, 10 min, 20 min, and 45 min). Results showed a significant inverted-U trend with exercise for 20 min resulting in optimal performance in a sample of young adults. However, there is no study as yet which has tested the effects of duration on a measure of task switching and in the older population

The present study fills a gap in the literature by characterizing the effect of acute moderate intensity aerobic exercise on the task switching aspect of executive function in older adults. Additionally, the dose-response relationship between exercise duration and task switching was explored to provide the foundation for exercise prescription in this specific population. We hypothesized that acute moderate intensity aerobic exercise would benefit task switching and that an inverted-U relationship between exercise duration and switching would be observed.

## 2. Methods

### 2.1. Participants

Forty-five older adults (mean age = 57.67 ± 5.06 years) were recruited from the local community, Taoyuan county and New Taipei city, via flyers and personal referrals. The participants met the following criteria: (1) aged from 55 to 65 years; (2) intact cognitive function defined as scoring >26 on the Mini-Mental State Examination (MMSE); (3) right-hand dominant; (4) normal vision and no color blindness; (5) no neurological or psychiatric disorders, and (6) no contraindications to exercise according to the Physical Activity Readiness Questionnaire (PAR-Q) [29].

All participants were asked to fill out a demographic sheet, to complete the International Physical Activity Questionnaire (IPAQ) [33], and to perform the Digit Span test of the Wechsler Adult Intelligence Scale (see Table 1). All participants provided written consent to participate after being informed of the potential risks. The protocol and consent forms were approved by the authorized Institutional Review Board of Fu-Jen Catholic University (FJU-IRB No: C104087). 

### 2.2. Task Switching Paradigm

A modified version of the task switching task, which has been previously used by Dai, Chang [34], was employed to examine the switching aspects of executive function. The task switching was generated by a computer program using Neuroscan Stim Software (version 2.0, Neuro Inc., El Paso, TX, USA) and was displayed on a 17-inch monitor.

All stimuli were presented as black single-digit numbers (1–9, but excluding 5; dimensions = 2 × 2 cm) with solid or dotted lines surrounding the number. Each stimulus was presented for 500 ms and separated by a 1500 ms inter-stimulus interval. All stimuli were focally presented in the center of a computer screen on a white background, the distance of which was 70 cm away from the participants. 

A total of six blocks involving four types of stimuli were included in the task switching task. The first and second blocks consisted of homogeneous conditions. In the first homogeneous block, the participants were required to determine whether the digit that was presented within solid lines was greater or less than 5 (i.e., stimulus A). In the second homogeneous block, the participants were asked to determine whether the digit that was presented within dotted lines was odd or even (i.e., stimulus B). Participants were required to respond to each stimulus by pressing the appropriate button (left or right) with their thumb as quickly and accurately as possible. Each homogeneous block consisted of 32 trials resulting in a total of 64 trials for the initial two blocks. 

The third to sixth blocks were four identical heterogeneous blocks. Stimuli in the heterogeneous condition were the same as were presented in the first and second homogeneous blocks (i.e., same digit number with solid or dotted lines). Participants were required to determine the magnitude when the digit was surrounded with solid lines or to identify the stimulus as odd/even when the digit was surrounded with dotted lines. The heterogeneous blocks consisted of consecutive trials (i.e., AABBAA), such that non-switch trials (i.e., the second A in an AA sequence or the second B in a BB sequence) and switch trials (i.e., the B in an AB sequence or the A in a BA sequence) could be identified. Each block consisted of 32 trials, resulting in 128 trials for these four blocks. 

Two main outcome variables from the four tasks conditions were analyzed: the global switching effects were assessed using performance during the homogeneous and heterogeneous conditions; and the local switching effects were assessed using performance on the two sub-conditions within the heterogeneous condition (i.e., non-switch and switch trials). Response time and accuracy were analyzed from each condition. The total testing time was approximately 25 min.

### 2.3. Cardiorespiratory Fitness Assessment 

Submaximal oxygen uptake (VO_2peak_) was determined using the YMCA cycle ergometry protocol [35] and was used to provide descriptive information about cardiorespiratory fitness [36]. The protocol, adopted from ACSM guidelines [29], was appropriate for adults who were determined to be in the Class A risk stratification [37].

Each individual’s fitness level was determined by completing three consecutive 3-min cycling stages using cycle ergometry (Lode, Excalibur Sport, Groningen, The Netherland), where heart rate (HR) was measured throughout. In the initial cycling stage, workload was set up as pedaling at a speed of 150 kpm min^−1^ (0.5 kg at 50 rpm) for 3 min. The test administrator recorded each participant’s HR during the final 15 to 30 s of both the second and third min. The HR in the first stage was utilized to determine the workload for the next two stages. For example, for participants with a HR of less than 80 bpm in the first stage, workload was increased to 750 kpm min^−1^ (2.5 kg at 50 rpm) for the second stage and 900 kpm min^−1^ (3.0 kg at 50 rpm) for the third stage. The VO_2peak_, was estimated by plotting HRs from the second and third stages and then observing VO_2_ at age-predicted maximal HR (206 − (0.67 × age)). 

### 2.4. Exercise Intensity Manipulation Check

HR: Exercise intensity was determined based upon age-predicted maximal HR. HR was assessed by short-range radio telemetry devices (Sport Tester PE 3000, Polar Electro Oy, Kempele, Finland) throughout the sessions. Two HR variables were used in analyses. Resting HR was defined as the average HR during baseline for each session. Treatment HR was operationalized as the average of HR measures taken at 2-min intervals during treatment. 

Rating of perceived exertion (RPE): Exercise intensity was also monitored using the subjective indicator of RPE. The Borg RPE scale [38], ranging from 6 to 20, was utilized to assess participants’ perceptions of effort during exercise. The anchors of 6 and 20 are equated to “no exertion” and “maximal exertion”, respectively. The test administrator recorded RPE at 2-min intervals during each exercise session and average RPE was computed.

### 2.5. Procedure

Participants were required to visit the laboratory individually on four separate occasions at approximately the same time of day, with at least 3 days between each visit. The sessions included three exercise sessions using the cycle ergometer (i.e., main exercise stage for 10, 20, or 45 min) and a control session (i.e., reading for 30 min). All participants experienced all four sessions presented in a counterbalanced order and they were instructed not to engage in any form of workout and not to drink any stimulating drinks within 12 h of study participation. 

On the first visit, eligible participants were screened for inclusion criteria and then completed the informed consent, the demographic sheet, the IPAQ, and the Digit Span test. The participant then conducted one of the four sessions. At the beginning of each session, participants were asked to sit quietly in a dimly lit room for 5–10 min to assess resting HR. Then, the participant was instructed to practice homogeneous blocks of 20 trials until 85% accuracy for the trial block was achieved. 

The participant was then instructed to conduct a selected session based upon their assigned order. The exercise session included a warm-up (5 min), the main exercise (i.e., duration based upon session), and cool-down (5 min) stages, and was designed following ACSM guidelines [29]. For the main exercise, participants were instructed to cycle at a moderate intensity (65% to 70% heart rate reserve, HRR) for the assigned duration. The specific exercise durations were selected to explore the dose-response relationship using a relatively short exercise period (10-min), a moderate duration exercise period that is commonly recommended in the exercise and cognition literature (20-min) [19,32], and a substantially longer exercise period (45-min). The control session required participants to sit quietly and read sport- or exercise-related magazines for 30 min. Following each session, the task switching test was performed by each participant. 

On the day of the control condition, participants completed the submaximal cardiovascular tests following the task switching task. This assessment was used to describe the fitness of the sample. Participants received $20 at the end of each session, and on the end of the fourth day, participants were told the detailed purpose and expectations of the study. 

### 2.6. Statistical Analysis

The sample size was determined from the effect size (partial η^2^ = 0.16) observed in a dose-response study of exercise duration and executive function [32], using a 2 by 4 repeated measures design, and with alpha = 0.05 and power = 0.8. Specifically, the effect size was selected because this is the only previous study that has examined the effects of exercise duration on executive function using a dose-response design and our study design was similar to that used in the previous study.

HR alterations among the four sessions (i.e., control, 10 min, 20 min, and 45 min) were analyzed using one-way repeated measures analysis of variance (RM ANOVA). RPE was analyzed using a one-way RM ANOVA to assess differences across the three exercise sessions (10 min, 20 min, and 45 min). Response time and accuracy for global switching were analyzed using a 4 (session) × (task condition: homogeneous and heterogeneous) RM ANOVA. Response time and accuracy for local switching were analyzed using a 4 (session) × (task condition: Switch and non-switch trials) RM ANOVA. A trend analysis was conducted for global and local switching (with the control condition equated to 0-min of exercise) to more closely examine the nature of the relationship between duration and performance. Greenhouse-Geisser corrections as well as paired-samples *t*-test with Bonferroni adjustments for multiple comparisons were applied. Partial η^2^ is presented as a measure of effect size for significant main and interaction effects. An alpha of 0.05 was considered significant.

## 3. Results

### 3.1. Exercise Intensity Manipulation Check

For HR, one-way RM ANOVA revealed a significant main effect for session such that the three exercise sessions resulted in significantly higher average HR (125.94 ± 11.00 bpm for 10 min, 128.29 ± 10.02 bpm for 20 min, and 127.60 ± 10.74 bpm for 45 min) as compared to the control session (72.87 ± 8.28 bpm) (*F* (3, 132) = 248.15, *p* < 0.01, partial η^2^ = 0.97), with no significant difference among the three exercise sessions (*p* > 0.05). For RPE, one-way RM ANOVA revealed a significant main effect for session, (*F* (2, 88) = 6.51, *p* < 0.02, partial η^2^ = 0.13), such that the 10 min exercise session resulted in significantly lower RPE (12.93 ± 2.00) as compared to 20 min (13.65 ± 1.77) and 45 min (13.67 ± 1.71).

### 3.2. Task Switching

Task switching performance across the four sessions and task conditions are summarized in Table 2.

**Figure 1:**
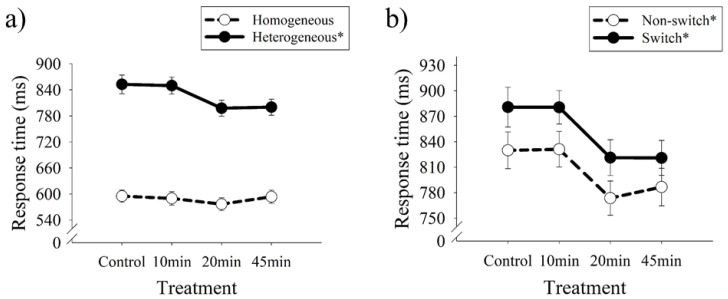
Dose-response relationship between exercise duration and task switching performance in response time. (**a**) Global switching; (**b**) Local switching. Note that lower scores (mean ± SEM) for response time represents better performance; * *p* < 0.05.

Global switching: Regarding response time, the two-way RM ANOVA revealed a significant main effect for session (*F* (3, 132) = 4.56, *p* < 0.01, partial η^2^ = 0.09), for task condition (*F* (1, 44) = 455.27, *p* < 0.01, partial η^2^ = 0.91), and for the interaction of session by task condition (*F* (3, 132) = 3.94, *p* < 0.01, partial η^2^ = 0.08). The subsequent analysis to decompose the interaction effect revealed that 20 min of exercise resulted in a shorter response time than control (*p* < 0.02) and 10 min (*p* < 0.05) sessions, but was not significantly different from 45 min in the heterogeneous condition; however, no significant differences were observed among control, 10 min, and 45 min. In the homogeneous condition, no significant differences among the four sessions were observed (*p* > 0.05, Figure 1a). The trend analysis demonstrated a linear trend for session (*F* = 8.84, *p* < 0.001) and also an interaction of session by task condition (*F* = 9.08, *p* < 0.01). When analyzed by task condition, the linear trend as a function of session was only evident for the heterogeneous condition (*F* = 11.58, *p* < 0.001) and was not evident for the homogeneous condition (*F* = 0.27, *p* > 0.05). A cubic trend for session was also observed (*F* = 4.19, *p* < 0.05).

Regarding accuracy, the analysis revealed a main effect for task condition (*F* (1, 44) = 5.80, *p* < 0.01, partial η^2^ = 0.11), with higher accuracy in the homogeneous condition than in the heterogeneous condition. However, no main effect for session or interaction of session by task condition was revealed. 

Local switching: Regarding response time, the two-way RM ANOVA revealed a significant main effect for session (*F* (3, 132) = 4.93, *p* < 0.01, partial η^2^ = 0.10) and for task condition (*F* (1, 44) = 16.26, *p* < 0.01, partial η^2^ = 0.27), but not for the interaction. The subsequent pairwise comparison for the main effect of session revealed that 20 min had shorter response time than control (*p* < 0.03) and 10 min (*p* < 0.03) sessions, but that 20 min was not significantly different from 45 min; however, no significant differences were evident between control, 10 min, and 45 min (Figure 1b). The subsequent pairwise comparison for the main effect of task condition revealed longer response time in the switch condition than in the non-switch condition (*F* = 12.26, *p* < 0.001). The trend analysis demonstrated a linear trend (*F* = 10.95, *p* < 0.001) and a cubic trend (*F* = 4.11, *p* < 0.05) as a function of session. Regarding accuracy, no significant main or interaction effects were observed.

## 4. Discussion

Despite the positive acute aerobic exercise effect on executive function, little is known about whether exercise affects the task switching aspect of executive function and the dose-response relationship between exercise duration and task switching in older adults has not been tested. In this study, older adults performed homogeneous and heterogeneous trial blocks following a control condition and 10, 20, and 45-min of moderate intensity exercise. Analyses of the results relative to global switching revealed that exercise duration did not affect performance in the homogeneous condition, but that acute moderate intensity aerobic exercise for 20 min resulted in shorter response times than control and 10 min sessions in the heterogeneous condition. Additionally, linear and cubic trends between exercise duration and global switching performance were revealed with faster times being predicted by longer duration exercise; however, performance following the 45-min session was not significantly different from the other three sessions. A similar pattern of better performance in the 20 min session and a linear and cubic relationship between exercise duration and response time were also observed for both types of trials (switch and non-switch) in local switching. In general, the results revealed that while exercise of longer duration, at least a 20 min session, improved task switching (i.e., linear trend), the effects of a 45 min session were the same as all other sessions.

In reviews of the literature, authors have concluded that acute exercise of at least 20 min [14] and between 20 to 60 min [39] would improve cognitive function. Our findings concur with these arguments, and further our understanding of this relationship by suggesting that 20-min of aerobic exercise at a moderate intensity benefits task switching in an aged population. The prescription of moderate intensity aerobic exercise for 20 min is frequently employed when examining cognitive function. Consistent with previous research that has examined other aspects of executive function including inhibition [19,20] and working memory [21,22], our findings support that this is an effective duration of moderate intensity exercise to improve task switching. While sharing some similarities, the subdomains of executive function do have distinguishing characteristics. For example, task switching is linked more to posterior regions of the dorsolateral prefrontal cortex and parietal regions [40]; whereas working memory engages more frontostriatal connections and basal ganglia [41]. These findings suggest the importance of examining the specific domain of executive function and indeed are compatible with the argument that exercise researchers should carefully consider the potentially task-specific effects of exercise on cognitive function [3]. Our acute exercise study focused on older adults because this population not only experiences age-related changes in physical function, but also has been less examined in the acute exercise literature [13]. Collectively, our findings suggest that acute aerobic exercise at a moderate intensity for 20 min has a positive effect on task switching in older adults. 

Notably, the dose-response relationships were revealed in the heterogeneous condition of global switching as well as in both non-switch and switch trials of local switching, but not in the homogeneous condition of global switching. Of the four conditions, the homogeneous condition involves the least cognitive effort (e.g., attention) because of consisting of a set of stimuli with only one attribute (e.g., odd or even). This is evidenced by the fact that participants performed dramatically and significantly faster in the homogeneous condition than in the heterogeneous condition. The next most difficult conditions are the non-switch trials in the heterogeneous condition, followed by the switch trials in the heterogeneous condition [25]. Our findings reflect that the positive effect of acute exercise on cognitive function is disproportionately larger in task conditions with greater executive function or cognitive demands. The selective improvement corresponds with findings of previous studies that have reported greater benefits following acute exercise in executive function task conditions requiring inhibition than were observed in conditions that required only basic information processing [42,43,44]. While the mechanisms for this observance of selective improvements in response to acute exercise is not yet understood, it is speculated that acute exercise alters brain activity that is specifically associated with executive function. For example, acute exercise was associated with shorter P3 latency, a neuroelectric index related to the classification, detection, and evaluation of a target stimulus [45], in incongruent (i.e., the condition associated with executive function) but not neutral conditions. This suggests that acute exercise serves to selectively improve cognitive processing speed during executive function [43,44]. Neuroimaging studies also demonstrate that acute exercise specifically alters brain activation under executive function conditions in left dorsolateral prefrontal cortex [27] as well as right middle frontal gyrus, right lingual gyrus, and left fusiform gyrus [46]. These findings provide the explanation regarding acute exercise related selective improvement from neurological and brain perspectives and warrant future investigation. 

The strengths of the present study include the examination of the dose-response relationship between a specific moderate intensity exercise and executive function, using a counterbalanced design with a relatively large sample. However, our results should be interpreted within the context of its limitations. The mean VO_2peak_ of our participants was 37.5 mL kg^−1^ min^−1^, suggesting that our healthy participants also had good levels of cardiovascular fitness. Given that older adults with higher fitness would be expected to receive greater benefits in cognitive function from acute exercise compared to those with lower fitness [19], the findings of this study may not generalize to unhealthy or low fit older adults. Furthermore, the exercise duration of 20 min may have been appropriate for this moderate intensity exercise, but the duration might need to be modified at other intensity levels. That being said, moderate intensity was chosen based upon both empirical evidence and practical concerns [13,14,30]. In addition, we only tested performance at three durations of exercise (10 min, 20 min, and 45 min) and within a limited range of durations (10 min to 45 min). Given the observed linear and cubic relationship between duration and performance, it will be important for future research to consider additional and more durations of exercise to more clearly identify the “sweet spot” relative to maximizing the beneficial effects and to confirm the nature of this cubic relationship. It seems likely that a quadratic or cubic effect might be observed with longer durations of exercise so that, in fact, increasingly longer durations of exercise do not result in increasingly better performance. 

One advantage of the current design is the consideration of exercise mode, intensity, and duration simultaneously. However, based upon the updated ACSM guidelines for exercise prescription, known as the FITT-VP principle (frequency, intensity, duration, type, volume, and progression), it is important to consider all of these components of an exercise prescription. Hence, volume, determined by both intensity and duration, is worth considering as we work to advance our understanding of the potential cognitive benefits of acute exercise in the future. Additionally, along with aerobic exercise, acute exercise studies have employed resistance exercise and have also observed a benefit to executive function [14,16,42]. Examination of these components will help to better characterize exercise prescription regarding executive function in the aged population and are worth consideration in future investigations. 

## 5. Conclusions

We employed a counterbalanced design with four sessions and a relatively large sample to explore the foundation of exercise prescription concerning mode, intensity, and duration relative to task switching performance in the older adults. A unique aspect of the study is, for the first time, the investigation of the dose response relationship between exercise duration and task switching in older adults. The exercise protocol was characterized according to ACSM guidelines, including a warm-up, a main exercise session, and a cool down providing higher ecological validity and clear implications for application. In general, acute aerobic exercise at moderate intensity for 20, but not 10 min, benefits task switching. A longer duration of exercise (45-min) was not distinguishable from either 10-min or 20-min of exercise. Hence, it appears that this duration is not optimal for benefiting task switching, but it also does not harm task switching and, hence, might be an option for the older adults interested in achieving other benefits that may require longer duration exercise. Continued examination of dose response relationships between specific exercise components and various cognitive domains is needed to better inform exercise prescription. 

## Figures and Tables

**Table 1:** Demographic characteristics for participants (mean ± SD).

Variable	Total
*n*	45
Female/Male (% Male)	26/19 (42%)
Age (Year)	57.67 ± 5.06
Height (cm)	163.04 ± 8.38
Weight (kg)	64.38 ± 13.09
Body mass index (kg m^−2^)	23.86 ± 3.74
Education (year)	14.11 ± 2.50
MMSE (Total score)	28.47 ± 1.79
Digit span (score) Forward Backward	15.29 ± 2.51 5.07 ± 2.09
Resting heart rate (bpm)	71.26 ± 8.84
IPAQ (METs wk^−1^)	1104.67 ± 1443.75
VO2peak (mL kg^−1^ min^−1^)	37.52 ± 9.92

Notes: MMSE = Mini-Mental State Examination; IPAQ = International Physical Activity Questionnaire.

**Table 2:** Response time and accuracy for global switching and local switching across four sessions (mean ± SD).

Variable	Session			
	Control	10 min	20 min	45 min
	Response time			
Global switching (ms)				
Heterogeneous	852.55 ± 144.21	849.72 ± 130.55	797.66 ± 124.16	800.10 ± 125.26
Homogeneous	594.96 ± 95.68	589.78 ± 103.47	576.46 ± 100.25	593.57 ± 100.86
Local switching (ms)				
Switch	880.74 ± 157.02	880.53 ± 132.17	821.28 ± 141.21	820.93 ± 138.35
Non-switch	829. 95± 144.83	831.14 ± 140.22	773.61 ± 134.42	786.61 ± 148.03
	Accuracy			
Global switching (%)				
Heterogeneous	94.01 ± 7.43	94.55 ± 7.47	93.55 ± 8.07	94.27 ± 9.42
Homogeneous	96.67 ± 5.60	96.49 ± 7.67	96.22 ± 5.63	95.76 ± 8.34
Local switching (%)				
Switch	94.23 ± 7.41	94.88 ± 8.50	93.83 ± 8.52	94.04 ± 9.68
Non-switch	94.69 ± 7.00	94.96 ± 8.46	94.32 ± 8.24	94.73 ± 9.59
